# Prevalence of dental caries and associated factors among primary school children: a population-based cross-sectional study in Riyadh, Saudi Arabia

**DOI:** 10.1186/s12199-018-0750-z

**Published:** 2018-11-30

**Authors:** Yazeed Abdullah Alhabdan, Abdulhameed Ghassan Albeshr, Nagarajkumar Yenugadhati, Hoda Jradi

**Affiliations:** 1 King Abdullah International Medical Research Center (KAIMRC), Riyadh, Saudi Arabia; 2 Department of Epidemiology and Biostatistics, College of Public Health and Health Informatics, King Saud bin Abdulaziz University for Health Sciences, Mail Code 2350, P.O. Box 22490, Riyadh, 11426 Saudi Arabia; 3 King Abdulaziz Medical City for National Guard, Ministry of National Guard, Riyadh, Saudi Arabia

**Keywords:** Dental caries, Primary teeth, Prevalence, Associated factors, Children

## Abstract

**Background:**

Dental caries is a preventable childhood disease, but public health efforts are hampered due to limited information on associated factors in vulnerable populations. Our study was aimed at estimating the prevalence of dental caries and identifying key associated factors in four major risk domains, including socioeconomic factors, child oral health behavior and practices, child feeding practices, and dietary habits among primary school children in Saudi Arabia.

**Methods:**

A cross-sectional study design was used to recruit 578 male Saudi primary school children, aged 6–8 years, from 12 primary schools in five different regions of Riyadh. Children were clinically screened to detect carious lesions in primary teeth according to World Health Organization’s criteria. Structured self-administered questionnaire was used to collect information on social and individual factors from the parents. The odds ratios and 95% confidence intervals of associated factors for dental caries were computed using logistic regression models; key factors were identified by systematic selection process that accounted for multicollinearity and bias correction.

**Results:**

Dental caries was prevalent among children (83%, 95% confidence interval 79.7–86.0%). Individual factors, including irregular brushing, late adoption of brushing habit, consulting dentist for symptomatic treatment, lack of breast feeding, sleeping with a bottle in mouth, habit of snacking between meals, low consumption of fruits, and frequent consumption of soft drinks and flavored milk, were predominantly associated with dental caries in children, instead of socioeconomic factors (*p* < 0.05, adjusted *R*-square 80%).

**Conclusion:**

Dental caries were prevalent in school children, and individual factors were predominantly associated with the disease.

**Electronic supplementary material:**

The online version of this article (10.1186/s12199-018-0750-z) contains supplementary material, which is available to authorized users.

## Introduction

Dental caries is a major oral health problem affecting 2.43 billion people (35.3% of the population) worldwide in the year 2010 [1]. A high burden of dental caries was evident among children in Saudi Arabia with an estimated prevalence of approximately 80% [2]; other high-risk areas include Latin America, Middle East, and South Asia [3]. The World Health Organization (WHO) emphasizes the need to reduce global burden of dental caries in attaining optimal health. Consequently, in the year 2003, WHO and Fédération Dentaire Internationale (FDI) World Dental Federation set global goals for oral health in 2020 to guide planners and policy makers to improve the status of oral health in their populations [4]. Unfortunately, knowledge gaps with respect to the availability of baseline data on oral health and population-specific key modifiable factors of dental caries restrict the ability of many developing nations and semi-developed countries, including Saudi Arabia to attain the goals set by WHO. In addition, competing interests in health care funding warrant prioritizing the associated factors to better direct public health mitigation efforts.

Although factors, such as occupational status, family income, and level of education of parents [5–9] that affect the socioeconomic status of populace, have been associated with dental caries, their relative impact on dental caries compared to individual factors is unclear. Moreover, modification of socioeconomic factors requires time-consuming macro level changes. In contrast, individual factors, such as child oral health behaviors, child feeding practices, and dietary habits that play a role in cariogenesis, could be targeted for modification by directing the limited resources to primary school children. Prior evidence illustrates the importance of adopting good oral health behaviors such as regular brushing of teeth, using mouthwash, and flossing teeth in reducing the disease burden and attaining optimal oral health [10]. Similarly, the role of sugary foods (e.g., candies) in cariogenesis was also well established [11]. However, the relative significance of aforementioned oral behavioral factors on cariogenesis compared to other host factors could vary significantly in different populations owing to cultural and behavioral practices.

In our globalized world, constant migration of individuals and transfer of certain behaviors or practices (e.g., favoring flavored milk over plain milk among children) [12] is the prevailing norm. Therefore, the knowledge of associated factors for dental caries in Saudi children not only benefit Saudi populace but also international organizations such as WHO and health authorities in directing the mitigation efforts at vulnerable populations (e.g., children).

This study aimed at estimating the prevalence of dental caries in primary teeth and identifying key associated factors in 6–8-year-old school children in Riyadh city of Saudi Arabia would contribute towards the knowledge of dental caries by enriching the baseline data and determining population-specific risk factors of such a highly prevalent and preventable condition. Our analysis is the first in Saudi Arabia to comprehensively evaluate and prioritize factors encompassing all four major risk domains for dental caries, including parental socioeconomic status, children oral health behavior and practices, child feeding practices, and dietary habits. In addition, the relative importance of individual factors (over socioeconomic factors) as determinants of dental caries was assessed using extensive modelling techniques.

## Methods

A population-based cross-sectional study design was employed to determine the burden of dental caries in primary teeth and key associated factors among 6–8-year-old male primary school children recruited from 12 government primary schools for boys located in 5 geographical regions (southern, northern, eastern, western and central) in Riyadh city, Saudi Arabia. The study included only Saudi nationals, whose parents were able to fill the self-administered questionnaire and provide informed consent for their child’s dental examination at school. Non-Saudi children or children with non-Saudi care givers or parents were excluded. This study was conducted in the year 2015 between September 1 and November 30.

### Sample size

Sample size was calculated using single proportion formula based on 95% confidence level, expected prevalence of 80% [2], precision of 0.05, and design effect of 2. The recommended sample size was 492 children with one of their parents as a single unit.

We anticipated low response rate owing to the outbreak of Middle East respiratory syndrome-corona virus (MERS-cov) in Riyadh city during the study period. Therefore, a total of 1130 questionnaires were distributed to parents and we received 578 completed questionnaires at a response rate of 51% from 12 out of 15 schools considered for recruitment among 513 government primary schools for boys in Riyadh region.

### Sampling technique

Our study sample was obtained by a multistage random sampling technique. Briefly, up to three primary schools in Riyadh were randomly selected from each of the five geographical locations in Riyadh city based on the list of primary schools obtained from the Ministry of Education. A maximum of 80 students were randomly selected from each of these schools. Each of the five regions in Riyadh city contributed a fifth of the total study sample.

### Screening for dental caries

Children underwent a simple dental examination based on the World Health Organization diagnostic criteria for oral health surveys [13]. The basic oral assessment of every child was performed by a single, well-trained professional dentist by seating each subject on a chair in a good day light using mouth mirror and dental probes. This simple oral examination poses no harm to children. The intra-examiner reliability was good based on re-examination of 30 children prior to the study (kappa value = 0.98). Caries status in the crown of primary teeth was assessed using decayed (d), missing (m), and filled (f) teeth (dmft) index [2, 13]; teeth missing (m) or filled (f) contributed to the overall dmft score only if they were missing or filled because of caries. A dmft score above null indicates the presence of caries, whereas a null score indicates the absence of caries [13].

### Parental questionnaire

A structured self-administered parental questionnaire was developed by relying on previous studies [13–20] and accounting for cultural sensitivities of the study population. The questionnaire was translated into Arabic and then back to English to ensure accuracy. Face validity, feasibility, and construct validity of the questionnaire was established prior to study.

The questionnaire responses provided data on age of the child, demographic and socioeconomic factors such as father’s education level, mother’s education level, parental occupation as health care provider, monthly income of the family, region of residence, type of residence, and availability of medical insurance with dental coverage. Parents also provided information on oral health behavior and practices of children, such as frequency of brushing teeth with toothpaste in a day; age at which children started brushing; use of dental floss; use of mouthwash; frequency of fluoride application; recent visit to the dentist; habit of eating after brushing teeth in night; and child feeding practices, such as type of milk feeding practice (breast-fed only/children mixed-fed with both breast milk and powdered milk/powdered milk only), age of child when breast feeding was stopped, age of child when bottle feeding was stopped, child sleeping with bottle in mouth, number of meals per day, number of snack items consumed between meals, and snack time corresponding to main meals (ate snacks with main meals only/ate snacks in between main meals or with main meals). Dietary information included use of multivitamin supplementation (no/yes) and consumption of fresh fruits, fresh vegetables or salads, fast food, candy, potato chips, sweetened chewing gum, fresh juice, flavored juices, soft drinks, fresh milk, and flavored milk at least twice a week (no/yes).

Some of the original variable categories were combined to create meaningful new groups, and facilitate appropriate analyses. In particular, the ‘frequency of brushing teeth’ variable was classified in to 3 categories (children brushing less than once daily/once daily/two times or more daily.

### Statistical analysis

All analyses of study data were performed using SAS software version 9.4 (SAS Institute Inc., Cary, NC, USA). Categorical variables were described as counts and percentages, whereas means and standard deviations (SD) were computed for continuous variables. The 95% confidence intervals for proportions were constructed using Clopper-Pearson exact tests. The independence of characteristics of study sample by caries status (presence or absence) was assessed using Pearson’s chi-squared test (or Fisher’s exact tests for smaller samples) and *p* values. Missing data were analyzed as a separate category (unknown or other) in corresponding variables.

The main associated factors for dental caries in our study were determined in three steps. In the first step, the association between each characteristic of study sample and the presence of dental caries was evaluated using univariate logistic regression analyses; all the variables that were significant at *p* value less than or equal to 0.05 were selected for second step of analyses. In the second step, the associated factors for dental caries among each of the four broader determinants of health, including socioeconomic factors, child oral health behavior and practices, child feeding practices, and dietary factors, were identified based on four separate stepwise logistic regression analyses. Subsequently, the covariates that were significant (*p* ≤ 0.05) in each of the four analyses were selected for further analysis. In the final step, a stepwise multivariate logistic regression analysis was performed on covariates selected from step two and variable age group of the child (6 or 7 or 8 years) to determine key associated factors for dental caries. In addition, multicollinearity was assessed using collinearity indices, eigenvalues, and variable decomposition proportions for all the multivariate models. One of the highly collinear variables was removed giving precedence to children oral health behavior and practice covariates. In addition, Firth’s bias correction was applied to the final multivariate model to address potential issues due to small sample size, and complete or quasi-complete separation.

The measures of association were reported as unadjusted odds ratios (uOR) and adjusted odds ratios (aOR) along with their corresponding 95% confidence intervals (95% CI). The discrimination, calibration and overall performance of the final multivariate model was assessed using concordance statistic, Hosmer and Lemeshow goodness-of-fit test, and adjusted Cox and Snell *R*-square, respectively. The performance of final model with and without socioeconomic factors was compared based on adjusted Cox and Snell *R*-square, which indicates the proportion of variation explained by the covariates in the model. Statistical analyses that yielded a *p* value less than or equal to 0.05 were considered significant.

## Results

A total of 578 primary school boys aged 6 to 8 years in Riyadh, Saudi Arabia, were analyzed in this study. The prevalence of dental caries in our sample was 83% (95% CI 79.7–86.0%). About 17% (95% CI 14.0–20.3%) of children had no carious lesions. The age-specific prevalence of dental caries among children aged 6, 7, and 8 years was 87.6% (95% CI 82.4–91.6%), 72.9% (95% CI 65.9–79.1%), and 88.4% (95% CI 82.7–92.8%), respectively. The mean age and dmft score in our sample was 6.92 (SD ± 0.82) and 4.20 (SD ± 2.96), respectively.

Table 1 provides the frequencies, percentages, and differences (by caries status) for various characteristics of study population. A significant number of fathers (65.7%, 95% CI 61.7–69.6%) and mothers (73.9%, 95% CI 70.1–77.4%) did not attend a college or university, and their children experienced high prevalence of dental caries. Majority of the children came from low-income families (59.7%, 95% CI 55.6–63.7%), and approximately 99% of them experienced dental caries. Most of the study subjects lived in rental homes, and 77% had no dental coverage in medical insurance. In general, the children had poor oral health behavior and practices as most of them started brushing at a late age (5 or more years) and brushed less than once daily (55%) in any given week. The use of dental floss and mouthwash was negligible, and most of the children visited a dentist for symptomatic treatment. Although the practice of breast feeding is common, most of the children were weaned by the first year. The practice of mixed feeding was common in our sample; approximately 81% of mixed-fed children experienced dental caries compared to 93% of children that were exclusively fed with either breast milk or powdered milk. The practice of sleeping with a bottle in mouth and frequent consumption of sugary snacks between meals was also common. The consumption of fresh fruits and fresh juice was less prevalent in our sample.

**Table 1:** Distribution of various characteristics of study population and the relationship with caries status

Characteristic	All subjects (*n* = 578)	Caries status		*p* value*
		Yes (*n* = 480)	No (*n* = 98)	
Age group				
6 years	217	190 (87.6)	27 (12.4)	<0.0001
7 years	188	137 (72.9)	51 (27.1)	
8 years	173	153 (88.4)	20 (11.6)	
I. Socioeconomic factors				
Father’s education				
Low level (high school or less)	380	368 (96.8)	12 (3.2)	< 0.0001
High level (college or university)	198	112 (56.6)	86 (43.4)	
Mother’s education				
Low level (high school or less)	427	405 (94.8)	22 (5.2)	< 0.0001
High level (college or university)	151	75 (49.7)	76 (50.3)	
Parent’s occupation				
Health care provider	30	2 (6.7)	28 (93.3)	< 0.0001
Other occupations	548	478 (87.2)	70 (12.8)	
Family monthly income				
Low (≤ 10,000SR)	345	341 (98.8)	4 (1.2)	< 0.0001
High (> 10,000SR)	233	139 (59.7)	94 (40.3)	
Region of residence				
North Riyadh	79	44 (55.7)	35 (44.3)	< 0.0001
South Riyadh	114	106 (93.0)	8 (7.0)	
East Riyadh	200	180 (90.0)	20 (10.0)	
West Riyadh	108	83 (76.9)	25 (23.1)	
Central Riyadh	77	67 (87.0)	10 (13.0)	
Type of residence				
Rental home	482	446 (92.5)	36 (7.5)	< 0.0001
Own home	96	34 (35.4)	62 (64.6)	
Medical insurance with dental coverage				
No	447	423 (94.6)	24 (5.4)	< 0.0001
Yes	131	57 (43.5)	74 (56.5)	
II. Child oral health behavior and practices				
Frequency of brushing teeth per day				
Less than once daily	320	318 (99.4)	2 (0.7)	< 0.0001
Once daily	128	100 (78.1)	28 (21.9)	
Two times or more daily	130	62 (47.7)	68 (52.3)	
Started brushing teeth at the age				
2 years or less	50	22 (44.0)	28 (56.0)	< 0.0001
3 years	82	46 (56.1)	36 (43.9)	
4 years	59	47 (79.7)	12 (20.3)	
5–6 years	333	315 (94.6)	18 (5.4)	
Unknown	54	50 (92.6)	4 (7.4)	
Dental floss use				
No	568	472 (83.1)	96 (16.9)	0.6806†
Yes	10	8 (80.0)	2 (20.0)	
Mouthwash use				
No	553	466 (84.3)	87 (15.7)	0.0011†
Yes	25	14 (56.0)	11 (44.0)	
Frequency of fluoride application				
None	454	414 (91.2)	40 (8.8)	< 0.0001
Every 6 months	42	18 (42.9)	24 (57.1)	
Every 1 year	38	8 (21.1)	30 (78.9)	
Unknown	44	40 (90.9)	4 (9.1)	
Recent visit to dentist				
Did not visit or unknown	99	71 (71.7)	28 (28.3)	< 0.0001
> 1 year	214	208 (97.2)	6 (2.8)	
≤ 1 year	265	201 (75.8)	64 (24.2)	
Reason for recent dental visit				
Did not visit or unknown	93	67 (72.0)	26 (28.0)	< 0.0001
Toothache	363	357 (98.3)	6 (1.7)	
Checkup or consultation	122	56 (45.9)	66 (54.1)	
Child ate after brushing teeth in the night				
No	221	133 (60.2)	88 (39.8)	< 0.0001
Yes	357	347 (97.2)	10 (2.8)	
III. Child feeding practices				
Type of milk feeding practice				
Breast-fed only	29	27 (93.1)	2 (6.9)	0.0163
Mixed-fed	487	395 (81.1)	92 (18.9)	
Powdered milk only	62	58 (93.6)	4 (6.5)	
Age of the child when breast feeding was stopped				
≤ 1 year	409	361 (88.3)	48 (11.7)	< 0.0001
> 1 year	169	119 (70.4)	50 (29.6)	
Age of the child when drinking with a bottle was stopped				
≤ 1 year	50	44 (88.0)	6 (12.0)	0.3286
> 1 year	528	436 (82.6)	92 (17.4)	
Child sleeps with bottle in mouth				
No	154	72 (46.8)	82 (53.2)	< 0.0001
Yes	424	408 (96.2)	16 (3.8)	
Number of meals per day				
1–2 meals	47	42 (89.4)	5 (10.6)	0.2286
3 or more meals	531	438 (82.5)	93 (17.5)	
Number of snacks consumed between meals				
One snack	156	78 (50.0)	78 (50.0)	< 0.0001
2 or more snacks	422	402 (95.3)	20 (4.7)	
Snack time corresponding to main meals				
With main meals only	80	26 (32.5)	54 (67.5)	< 0.0001
Between or with main meals	390	370 (94.9)	20 (5.1)	
Unknown	108	84 (77.8)	24 (22.2)	
IV. Dietary factors (consumed at least twice a week)				
Multivitamin supplementation				
No	532	446 (83.8)	86 (16.2)	0.0853
Yes	46	34 (73.9)	12 (26.1)	
Fresh fruits				
No	408	390 (95.6)	18 (4.4)	< 0.0001
Yes	170	90 (52.9)	80 (47.1)	
Fresh vegetables or salads				
No	137	127 (92.7)	10 (7.3)	0.0006
Yes	441	353 (80.0)	88 (20.0)	
Fast food				
No	235	157 (66.8)	78 (33.2)	< 0.0001
Yes	343	323 (94.2)	20 (5.8)	
Candy				
No	96	60 (62.5)	36 (37.5)	< 0.0001
Yes	482	420 (87.1)	62 (12.9)	
Potato chips				
No	96	64 (66.7)	32 (33.3)	< 0.0001
Yes	482	416 (86.3)	66 (13.7)	
Sweetened chewing gum				
No	467	379 (81.2)	88 (18.8)	0.0131
Yes	111	101 (91.0)	10 (9.0)	
Fresh juice				
No	394	370 (93.9)	24 (6.1)	< 0.0001
Yes	184	110 (59.8)	74 (40.2)	
Flavored juices				
No	58	44 (75.9)	14 (24.1)	0.1243
Yes	520	436 (83.8)	84 (16.2)	
Soft drinks				
No	183	105 (57.4)	78 (42.6)	< 0.0001
Yes	395	375 (94.9)	20 (5.1)	
Fresh milk				
No	244	220 (90.2)	24 (9.8)	< 0.0001
Yes	334	260 (77.8)	74 (22.2)	
Flavored milk				
No	372	294 (79.0)	78 (21.0)	0.0006
Yes	206	186 (90.3)	20 (9.7)	

*p value was based on the Pearson chi-squared test to evaluate the independence of sample characteristic and caries status

†*p* value was based on Fisher’s exact tests

The summary of variables selected during different steps of selection process is illustrated in Table 2. Barring few exceptions, almost all the factors were significantly associated with dental caries’ experience in univariate analyses (step 1). In the ensuing step 2 multivariate analysis, a limited number of factors were associated with dental caries in each of the four risk domains with more concessions observed among dietary factors. In the final step of model selection, the highly collinear child feeding covariate (i.e., age of the child when breast feeding was stopped) was excluded to address multicollinearity. Our model selection process yielded 12 variables that were significant at *p* < 0.05 for inclusion in the final model. Although association measures were not provided in Table 2 to avoid confusion, interested readers could find these details in Additional file 1.

**Table 2:** Summary of variables selected in four main risk domains, encompassing socioeconomic factors, child oral health behavior and practices, child feeding practices, and dietary factors, at different steps of model selection process using logistic regression (LR) analysis*

All variables	Step 1 univariate LR within risk domain	Step 2 multivariate LR within risk domain	Final step multivariate LR all 4 risk domains
I. Socioeconomic factors			
Father’s education	X	–	–
Mother’s education	X	X	X
Parent’s occupation	X	X	–
Family monthly income	X	X	X
Region of residence	X	X	–
Type of residence	X	–	–
Medical insurance with dental coverage	X	X	X
II. Child oral health behavior and practices			
Frequency of brushing teeth per day	X	X	X
Started brushing teeth at the age	X	X	X
Dental floss use	–	–	–
Mouthwash use	X	–	–
Frequency of fluoride application	X	X	–
Recent visit to dentist	X	–	–
Reason for recent dental visit	X	X	X
Child ate after brushing teeth in the night	X	–	–
III. Child feeding practices			
Type of milk feeding practice	X	X	X
Age of the child when breast feeding was stopped	X	X	†
Age of the child when drinking with a bottle was stopped	–	–	–
Child sleeps with bottle in mouth	X	X	X
Number of meals per day	–	–	–
Number of snacks consumed between meals	X	X	X
Snack time corresponding to main meals	X	X	–
IV. Dietary factors (consumed at least twice a week)			
Multivitamin supplementation	–	–	–
Fresh fruits	X	X	X
Fresh vegetables or salads	X	–	–
Fast food	X	–	–
Candy	X	–	–
Potato chips	X	–	–
Sweetened chewing gum	X	–	–
Fresh juice	X	X	–
Flavored juices	–	–	–
Soft drinks	X	X	X
Fresh milk	X	–	–
Flavored milk	X	X	X

*The variables selected in the step were marked with an “X,” and variable excluded is marked as “–.” The variables selected were significant at *p* value less than or equal to 0.05

†The variable “Age of the child when breast feeding was stopped” was excluded to address the issue of collinearity in the final model

The unadjusted and adjusted odds ratios along with their 95% confidence intervals (based on Firth’s bias correction) for the variables, representing all four risk domains, in the final model are reported in Table 3. It should be noted that factors representing low socioeconomic status, such as low level of maternal education, low family income, and lack of dental insurance, were associated with a minimum of fourfold increased dental caries experience. Child oral health practices, such as failure to brush teeth at least once a day, failure to start brushing on or before a child attained 2 years of age, and visiting dentist for symptomatic treatment, were associated with dental caries experience in children. Children habituated to sleeping with bottle in mouth experienced 4.4-fold higher dental caries compared to children not practicing this habit (aOR = 4.4, 95% CI 1.4–13.4). In addition, lack of mixed feeding and consuming two or more sugary snack items between meals were predominantly associated with dental caries experience (*p* < 0.05). Dietary habits, such as less consumption of fresh fruits and frequent consumption of soft drinks and flavored milk, were significantly associated with dental caries with an odds ratio of 11.6, 5.3, and 7.7, respectively. The final model was well calibrated (*p* = 0.7667; Hosmer and Lemeshow goodness-of-fit test) with very high discriminatory power (*c*-statistic = 99%) and high overall performance (adjusted *R*-square of 88%).

**Table 3:** Unadjusted odds ratios (uOR), adjusted odds ratios (aOR), and their respective 95% confidence intervals (95% CI) of the key associated factors for dental caries in primary school children aged 6–8 years

Characteristic	Unadjusted odds ratio		Adjusted odds ratio*	
	uOR	95% CI	aOR	95% CI
I. Socioeconomic factors				
Mother’s education				
Low level (high school or less)	18.7	10.9–31.8	4.4	1.3–15.5
High level (college or university)	1.0	(Ref)†	1.0	(Ref)†
Family monthly income				
Low (≤ 10,000SR)	57.6	20.8–159.7	28.2	5.2–153.9
High (> 10,000SR)	1.0	(Ref)†	1.0	(Ref)†
Medical insurance with dental coverage				
No	22.9	13.4–39.2	4.2	1.2–14.3
Yes	1.0	(Ref)†	1.0	(Ref)†
II. Child oral health behavior and practices				
Frequency of brushing teeth per day				
Less than once daily	174.4	41.6–730.3	30.1	3.1–294.3
Once daily	3.9	2.3–6.7	0.2	0.0–0.6
Two times or more daily	1.0	(Ref)†	1.0	(Ref)†
Started brushing teeth at the age				
2 years or less	1.0	(Ref)†	1.0	(Ref)†
3 years	1.6	0.8–3.3	21.2	2.2–203.6
4 years	5.0	2.1–11.6	9.3	1.3–68.0
5–6 years	22.3	10.7–46.4	1.7	0.3–9.6
Unknown	15.9	5.0–50.8	0.1	0.0–1.3
Reason for recent dental visit				
Did not visit or unknown	3.0	1.7–5.4	0.1	0.0–0.6
Toothache	70.1	29.0–169.4	21.4	3.9–119.3
Checkup or consultation	1.0	(Ref)†	1.0	(Ref)†
III. Child feeding practices				
Type of milk feeding practice				
Breast-fed only	3.1	0.7–13.5	33.1	4.7–231.4
Mixed-fed	1.0	(Ref)†	1.0	(Ref)†
Powdered milk only	3.4	1.2–9.5	38.4	3.2–459.9
Child sleeps with bottle in mouth				
No	1.0	(Ref)†	1.0	(Ref)†
Yes	29.0	16.1–52.5	4.4	1.4–13.4
Number of snacks consumed between meals				
One snack	1.0	(Ref)†	1.0	(Ref)†
2 or more snacks	20.1	11.6–34.8	6.8	2.1–21.4
IV. Dietary factors (consumed at least twice a week)				
Fresh fruits				
No	19.3	11.0–33.7	11.6	2.8–48.2
Yes	1.0	(Ref)†	1.0	(Ref)†
Soft drinks				
No	1.0	(Ref)†	1.0	(Ref)†
Yes	13.9	8.1–23.8	5.3	1.5–18.0
Flavored milk				
No	1.0	(Ref)†	1.0	(Ref)†
Yes	2.5	1.5–4.2	7.7	2.6–23.0

*Key associated factors for dental caries selected using stepwise multivariate logistic regression model in the final step of model selection process were included in the final model, and Firth’s bias correction was applied

†Reference category for the variable

Subsequent exclusion of three variables representing socioeconomic status from the final model also resulted in a well-calibrated model (*p* = 0.3502; Hosmer and Lemeshow goodness-of-fit test) with very high discriminatory power (*c*-statistic = 98%). However, a slight reduction in overall performance from 88 to 80% was noted, signifying the influence of individual or personal factors (represented in the remaining three risk domains) on dental caries experience in children; the overall performance of model with variables representing socioeconomic status was 59%. In addition, the higher magnitude of adjusted odds ratios of individual factors (ranging from 4.4 to 38.4) compared to aORs of socioeconomic factors (ranging from 4.2 to 28.2) and the lower confidence limits that were consistently above 1.5 lend further support to the predominant influence of individual factors on dental caries’ experience in children.

## Discussion

Dental caries was prevalent among 6- to 8-year-old primary school children in Saudi Arabia (83%, 95% CI 79.7–86.0). We identified individual factors, encompassing three major risk domains (children oral health behavior and practices, child feeding practices, and dietary habits) that were predominantly associated with dental caries’ experience in our study. Especially, child oral health behavior and practices, such as brushing teeth at least once daily, starting the practice of brushing earlier than 2 years, and visiting a dentist regularly, were significantly associated with dental caries. In addition, children mixed-fed with both breast milk and powdered milk, children sleeping with bottle in mouth, and the practice of snacking two or more items between meals were linked to dental caries experience in children. Dietary habits, such as less frequent consumption of fresh fruits (once a week or less) and more frequent consumption of soft drinks and flavored milk (more than once a week), were significantly associated with dental caries in our study. In our sample, socioeconomic factors (less-educated mothers, low family income, and lack of dental insurance coverage) were less influential than individual factors in determining dental caries’ experience in 6–8-year-old male primary school children.

The high prevalence of dental caries observed among primary school children in our sample was consistent with previous studies in Saudi Arabia [14, 21–24] and UAE [25]. A recent meta-analysis of various dental caries studies in different regions of Saudi Arabia determined the prevalence to be 80% [2]. Furthermore, the observed prevalence of dental caries among children in the present study was substantially higher than the target established for the year 2000 (50%) by WHO/FDI [26]. The collective evidence from our study and previous studies confirm the endemic nature of dental caries in Middle Eastern population and signify the burden on public health.

It is interesting to note that dental caries’ experience among primary school children was better explained by individual factors (80%) rather than socioeconomic factors (59%) in our study, which is consistent with weaker role of socioeconomic factors observed in developed nations [6, 15]. This notion was further supported by the relatively stronger associations observed between individual factors and dental caries experience in our study. In contrast, several cross-sectional and longitudinal studies from developing nations demonstrated the dominant role of socioeconomic factors in dental caries’ experience [18, 27–29]. The risk profile of dental caries among children in Saudi Arabia appears to follow the theme in developed world, where oral health behavioral practices and dietary habits were relatively more important [30]. However, efforts directed at improving socioeconomic status should be continued, owing to evidence from the present study and prior studies that identified maternal education and family income as consistent associated factors for dental caries [16, 17, 27, 28]. In addition, the availability of dental coverage in medical insurance was associated with dental caries. Although literary evidence was inconsistent in Saudi Arabia [16], the alarming proportion of children (77%) that lacked dental coverage in medical insurance warrant further attention.

Our results were consistent with previous studies on dental caries that reported an association between dental caries and good oral health behaviors in general [10, 19], and tooth brushing habits in particular [31, 32]. A recent meta-analysis identified a 1.5-fold higher risk of dental caries among people brushing less than once daily compared to those brushing regularly (odds ratio (OR) = 1.56; 95% CI 1.37–1.78) [33]. An overwhelming majority of children started brushing after 2 years (82%, 95% CI 78.6–85.1%) in the present study, consistent with late adoption of brushing observed in previous studies in Saudi Arabia [20, 34] and in Philippines [35]. However, the higher risk of dental caries observed uniquely among children who started brushing late at 3 or 4 years, in our sample, warrant further investigation. Particularly, future studies could evaluate the possible role of cultural habit of using chewing stick (Miswak) for cleaning teeth on better outcome observed among children starting brushing at ages 5–6 compared to those starting brushing at 3 or 4 years in Saudi Arabia. Given the importance of brushing teeth regularly and mouthwash use in maintaining good oral hygiene and preventing dental caries [10], and lower prevalence of these habits observed in our study, detailed investigation of various brushing practices (e.g., use of fluoridated/non fluoridated toothpaste, and use of chewing stick for cleaning teeth), and other oral hygiene practices (e.g., use of fluoride containing mouthwash) among primary school children in Saudi Arabia is necessary. Furthermore, interventions aimed at encouraging good oral health behaviors among children should be undertaken.

The negative attitude or apprehension towards visiting a dentist was clearly evident in our study, where only 21.1% (95% CI 17.8–24.7%) of children visited a dentist for regular check-up, while the others visited for symptomatic treatment (e.g., toothache). The problem was even worse among younger children in Saudi Arabia; a mere 11% of children visited dentist for regular checkup on their first visit [20]. This dangerous trend might have prevented patients from availing sound advice on preventive oral health practices, thereby contributing to high prevalence, delayed recognition, and management of dental caries in Saudi Arabia. Therefore, Saudi children would benefit from publicly funded school-based dental screening programs that aid in timely detection and management of dental and other oral health problems. In addition, regular dental screening programs targeted at school children have an added benefit of realizing cost savings due to reduced need for advanced dental care [36].

The present study found a 4.5 (OR = 4.5, 95% CI 1.5–13.8)-fold higher risk of dental caries among children falling asleep with the bottle in their mouth, which was consistent with literary evidence [37–39]. However, the magnitude of risk among Australian children sleeping with a bottle in mouth was much lower (OR = 1.5, 95% CI = 1.1–2.2) [39]. It was suggested that decreased salivary flow and reduced swallowing reflex as the child gets drowsier would allow carbohydrates to remain in the mouth and pool around the teeth priming the area for bacterial attack [40, 41].

The practice of frequently consuming sugary snacks between meals was associated with dental caries in our study. However, current evidence has been inconsistent with some studies indicating a positive association [42, 43], while others failed to observe such a relationship [44]. Therefore, further evaluation and confirmation of this globally relevant predictor is warranted.

Although breast feeding is commonly practiced in Western countries [45, 46], the practice of mixed feeding or partial breast feeding (with breast milk and powdered milk) was predominant in Saudi Arabia [47, 48]. Children in our study that were never breast-fed had higher risk of caries, which was consistent with existing literature [49–51]. Breast milk by itself was not cariogenic [52], but the reported cariogenicity of certain infant formulas [53] and a higher risk associated with practice of breast feeding until late infancy (> 12 months - OR = 1.99; 95% CI 1.35–2.95) [54] should not discourage the practice of mixed feeding until the emergence of new evidence. Interestingly, children in our study that were exclusively breast-fed also experienced higher risk of caries, rendering support to the practice of mixed feeding. As noted in previously published literature [54], it is possible that the practice of breast feeding until late infancy could have played a role in excess risk observed in Saudi children; however, further research based on a larger sample is warranted to confirm our findings and determine the role of duration of exclusive breast feeding on caries risk among children in Saudi Arabia.

Furthermore, our study identified that eating patterns and food choices play an important role in dental caries experience in children. Interestingly, the observed association between flavored milk and dental caries in this study could be a result of evolving trends in milk consumption practices in Saudi Arabia. Although prior observational studies [55, 56] contrast our findings, a moderate cariogenic potential of flavored milk observed in a recent animal experiment and the possibility of developing nations adopting this new trend warrant further evaluation [57]. Incidentally, our study contributed towards ever increasing evidence for the association between dental caries and sodas (or soft drinks) [58–60]. The acidic content of these soft drinks combined with sugars were known to reduce oral pH and increase the cariogenic potential of tooth [61].

It is noteworthy that low consumption of fresh fruits (less than twice a week) was associated with increased risk of dental caries among primary school children in this study. In contrast, the literary evidence did not provide a clear benefit of eating fresh fruits in preventing cariogenesis [62, 63]. However, certain fruit extracts (e.g., *Morinda citrifolia*) have been associated with inhibiting the growth of cariogenic bacteria [64], indicating the need to further evaluate the relevance of fresh fruit consumption to dental caries experience. In general, our findings were consistent with studies that linked intake of foods with high sugar content and dental caries in Saudi Arabia [19, 65] and other places [58–60, 66].

The strengths of this study are multi-fold. Information from various risk domains was systematically analyzed to aid in prioritizing the modifiable factors associated with dental caries experience in children. Unlike several prior studies in this area [10, 14, 16, 19, 34], this study addressed the issue of multicollinearity and corrected potential bias from small sample in the analysis. The comprehensive nature of information collected encompassing various risk domains enabled us to evaluate the relative importance of individual factors over socioeconomic factors, a component seldom addressed in previous studies. Our study provides much needed baseline statistics on several population characteristics to aid not only local authorities, but also international organizations (e.g., WHO) to evaluate and improve the health programs aimed at mitigating the burden of dental caries in children.

However, certain limitations of this study should be considered while interpreting the results. A self-administered questionnaire was used as the main study instrument, which is subjected to recall bias. However, we do not expect our results to be grossly affected by recall, owing to recurrent and current themes tested in the questionnaire. For example, we would expect a more accurate recollection of tooth brushing habits and child feeding practices that were routine activities performed in the recent past; collection of information on flavored milk, a recent trend in Saudi Arabia, serves as an example for current themes. The study sample was restricted to 6–8-year-old male primary school children in Riyadh city of Saudi Arabia, which warrants caution in generalizing the results to the entire country; however, given the cultural homogeneity and urbanity of the area, we would expect our estimates to be relevant to general population. Our study does not support generalizing the results to girl children, as our sample was restricted to boys to comply with school regulations and cultural sensitivities of Saudi population. Although some of our findings could be relevant to girls owing to shared cultural practices, future research should evaluate and confirm gender-related differences. Moreover, the cross-sectional nature of this study warrants against drawing causal inferences.

## Conclusion

The burden of dental caries is high in Saudi Arabia with eight out of ten primary school children aged 6–8 years suffering from this preventable condition. Several individual factors encompassing three risk domains, including oral health behaviors and practices, child feeding practices, and dietary habits, were found to be more relevant factors associated with dental caries than socioeconomic factors. Our results were consistent with findings in developed world where poor brushing habits, lack of dental coverage in health insurance, and high consumption of sodas were predominantly associated with dental caries. Future research should focus on confirming some of the unique or globally relevant associated factors for dental caries identified in our study, including late adoption of brushing, frequent consumption of sugary snacks between meals, and consumption of fresh fruits and flavored milk. Our results support the development and implementation of public awareness campaigns or health education programs targeted at primary school children to promote good oral health behaviors, feeding practices, and dietary habits.

## Additional file

Additional file 1: **Table S1.** Unadjusted odds ratios (uOR), adjusted odds rations for variables selected within each risk domain (dOR), and adjusted odds ratios (aOR) for variables selected from all four risk domains at different steps of model selection process*. (DOCX 34 kb)

## Abbreviations

95% CI: 95% confidence interval
aOR: Adjusted odds ratio
dmft index: Decayed, missing and filled teeth index
FDI: Fédération Dentaire Internationale
MERS-cov: Middle East respiratory syndrome-corona virus
OR: Odds ratio
SD: Standard deviation
uOR: Unadjusted odds ratio
WHO: World Health Organization
