# Supplementary material for: Prevalence of dental caries and associated factors among primary school children: a population-based cross-sectional study in Riyadh, Saudi Arabia
Source: Environ Health Prev Med. 2018 Nov 30;23:60. doi: 10.1186/s12199-018-0750-z (PMC6267843; doi:10.1186/s12199-018-0750-z)
Supplement: Supplementary file 1 — Table S1. Unadjusted odds ratios (uOR), adjusted odds rations for variables selected within each risk domain (dOR), and adjusted odds ratios (aOR) for variables selected from all four risk domains at different steps of model selection process*. (DOCX 34 kb) [file 12199_2018_750_MOESM1_ESM.docx]

**Additional file 1 for “Prevalence of dental caries and associated factors among primary school children: a population based cross-sectional study in Riyadh, Saudi Arabia.”**

As an extension to Table 2 in the main paper, Table S1 provides odds ratios and 95% confidence intervals for the variables selected during the model selection process. Specifically, unadjusted odds ratios (uOR), adjusted odds rations for variables selected with in each risk domain (dOR), and adjusted odds ratios (aOR) for variables selected from all four risk domains were reported along with their 95% confidence intervals for the relationship between various characteristics of study population and dental caries.

**Table S1** Unadjusted odds ratios (uOR), adjusted odds rations for variables selected within each risk domain (dOR), and adjusted odds ratios (aOR) for variables selected from all four risk domains at different steps of model selection process*

| **Characteristic** | **Unadjusted odds ratio** | | **Adjusted odds ratio within each risk domain** | | **Final adjusted odds ratios across all risk domains** | |
| --- | --- | --- | --- | --- | --- | --- |
|  | **uOR** | **95% CI** | **dOR** | **95% CI** | **aOR** | **95% CI** |
|  |  |  |  |  |  |  |
| **I. Socioeconomic factors:** |  |  |  |  |  |  |
|  |  |  |  |  |  |  |
| **Father's education** |  |  |  |  |  |  |
| Low level (High school or less) | 23.5 | 12.4–44.6 | - | - | - | - |
| High level (College or University) | 1.0 | (Ref)† |  |  |  |  |
| **Mother's Education** |  |  |  |  |  |  |
| Low level (High school or less) | 18.7 | 10.9–31.8 | 3.8 | 1.9–7.7 | 4.4 | 1.3–15.5 |
| High level (College or University) | 1.0 | (Ref)† | 1.0 | (Ref)† | 1.0 | (Ref)† |
| **Parent's occupation** |  |  |  |  |  |  |
| Health care provider | 1.0 | (Ref)† | 1.0 | (Ref)† | - | - |
| Other occupations | 95.6 | 22.3–410 | 10.2 | 2.1–48.8 |  |  |
| **Family monthly income** |  |  |  |  |  |  |
| Low (≤10000SR) | 57.6 | 20.8–159.7 | 10.8 | 3.5–33.2 | 28.2 | 5.2–153.9 |
| High (> 10000SR) | 1.0 | (Ref)† | 1.0 | (Ref)† | 1.0 | (Ref)† |
| **Region of residence** |  |  |  |  |  |  |
| North Riyadh | 1.0 | (Ref)† | 1.0 | (Ref)† | - | - |
| South Riyadh | 10.5 | 4.5–24.5 | 8.9 | 2.7–29.7 |  |  |
| East Riyadh | 7.2 | 3.8–13.6 | 4.0 | 1.6–9.99 |  |  |
| West Riyadh | 2.6 | 1.4–5 | 3.4 | 1.4–8.5 |  |  |
| Central Riyadh | 5.3 | 2.4–11.9 | 4.3 | 1.3–13.6 |  |  |
| **Type of residence** |  |  |  |  |  |  |
| Rental home | 22.6 | 13.2–38.7 | - | - | - | - |
| Own home | 1.0 | (Ref)† |  |  |  |  |
| **Medical insurance with dental coverage** |  |  |  |  |  |  |
| No | 22.9 | 13.4–39.2 | 5.0 | 2.6–9.8 | 4.2 | 1.2–14.3 |
| Yes | 1.0 | (Ref)† | 1.0 | (Ref)† | 1.0 | (Ref)† |
|  |  |  |  |  |  |  |
| **II. Child oral health behavior and practices:** |  |  |  |  |  |  |
|  |  |  |  |  |  |  |
| **Frequency of brushing teeth per day** |  |  |  |  |  |  |
| Less than once daily | 174.4 | 41.6–730.3 | 69.2 | 9.8–487.2 | 30.1 | 3.1–294.3 |
| Once daily | 3.9 | 2.3–6.7 | 1.6 | 0.8–3.3 | 0.2 | 0.0–0.6 |
| Two times or more daily | 1.0 | (Ref)† | 1.0 | (Ref)† | 1.0 | (Ref)† |
| **Started brushing teeth at the age:** |  |  |  |  |  |  |
| 2 years or less | 1.0 | (Ref)† | 1.0 | (Ref)† | 1.0 | (Ref)† |
| 3 years | 1.6 | 0.8–3.3 | 5.1 | 1.7–15.4 | 21.2 | 2.2–203.6 |
| 4 years | 5.0 | 2.1–11.6 | 5.7 | 1.8–17.7 | 9.3 | 1.3–68.0 |
| 5 - 6 years | 22.3 | 10.7–46.4 | 4.8 | 1.6–14.5 | 1.7 | 0.3–9.6 |
| Unknown | 15.9 | 5.0–50.8 | 0.5 | 0.1–3.1 | 0.1 | 0.0–1.3 |
| **Dental floss use** |  |  |  |  |  |  |
| No | 1.2 | 0.3–5.9 | - | - | - | - |
| Yes | 1.0 | (Ref)† |  |  |  |  |
| **Mouthwash use** |  |  |  |  |  |  |
| No | 4.2 | 1.9–9.6 | - | - | - | - |
| Yes | 1.0 | (Ref)† |  |  |  |  |
| **Frequency of fluoride application** |  |  |  |  |  |  |
| None | 38.8 | 16.7–90.3 | 8.6 | 2.7–28.0 | - | - |
| Every six months | 2.8 | 1.0–7.6 | 5.4 | 1.5–19.3 |  |  |
| Every one year | 1.0 | (Ref)† | 1.0 | (Ref)† |  |  |
| Unknown | 37.5 | 10.3–136.3 | 24.9 | 5.5–112.0 |  |  |
| **Recent visit to dentist** |  |  |  |  |  |  |
| Did not visit or unknown | 0.8 | 0.5–1.4 | - | - | - | - |
| > 1 year | 11.0 | 4.7–26.1 | - | - |  |  |
| ≤ 1 year | 1.0 | (Ref)† |  |  |  |  |
| **Reason for recent dental visit** |  |  |  |  |  |  |
| Did not visit or unknown | 3.0 | 1.7–5.4 | 0.8 | 0.3–2.0 | 0.1 | 0.0–0.6 |
| Toothache | 70.1 | 29.0–169.4 | 15.9 | 5.3–47.6 | 21.4 | 3.9–119.3 |
| Checkup or consultation | 1.0 | (Ref)† | 1.0 | (Ref)† | 1.0 | (Ref)† |
| **Child ate after brushing teeth in the night** |  |  |  |  |  |  |
| No | 1.0 | (Ref)† | - | - | - | - |
| Yes | 23.0 | 11.6–45.5 |  |  |  |  |
|  |  |  |  |  |  |  |
| **III. Child feeding practices:** | | | |  |  |  |
|  |  |  |  |  |  |  |
| **Type of milk feeding practice** |  |  |  |  |  |  |
| Breast-fed only | 3.1 | 0.7–13.5 | 16.5 | 3.3–81.9 | 33.1 | 4.7–231.4 |
| Mixed-fed | 1.0 | (Ref)† | 1.0 | (Ref)† | 1.0 | (Ref)† |
| Powdered milk only | 3.4 | 1.2–9.5 | 3.9 | 1.1–13.9 | 38.4 | 3.2–459.9 |
| **Age of the child when breast feeding was stopped** |  |  |  |  |  |  |
| ≤ 1 year | 3.2 | 2.0–4.9 | 2.4 | 1.2–4.9 | †† | †† |
| > 1 year | 1.0 | (Ref)† | 1.0 | (Ref)† |  |  |
| **Age of the child when drinking with a bottle was stopped** |  |  |  |  |  |  |
| ≤ 1 year | 1.5 | 0.6–3.7 | - | - | - | - |
| > 1 year | 1.0 | (Ref)† |  |  |  |  |
| **Child sleeps with the bottle in mouth** |  |  |  |  |  |  |
| No | 1.0 | (Ref)† | 1.0 | (Ref)† | 1.0 | (Ref)† |
| Yes | 29.0 | 16.1–52.5 | 12.2 | 6.1–24.5 | 4.4 | 1.4–13.4 |
| **Number of meals per day** |  |  |  |  |  |  |
| 1 - 2 meals | 1.0 | (Ref)† | - | - | - | - |
| 3 or more meals | 1.8 | 0.7–4.6 |  |  |  |  |
| **Number of snacks consumed between meals** |  |  |  |  |  |  |
| One snack | 1.0 | (Ref)† | 1.0 | (Ref)† | 1.0 | (Ref)† |
| 2 or more snacks | 20.1 | 11.6–34.8 | 4.9 | 2.4–9.8 | 6.8 | 2.1–21.4 |
| **Snack time corresponding to main meals** |  |  |  |  |  |  |
| With main meals only | 1.0 | (Ref)† | 1.0 | (Ref)† | - | - |
| Between or with main meals | 38.4 | 20.1–73.5 | 4.7 | 1.9–11.3 |  |  |
| Unknown | 7.3 | 3.8–13.9 | 2.5 | 1.1–5.9 |  |  |
|  |  |  |  |  |  |  |
| **IV. Dietary factors:**  (consumed at least twice a week) | |  |  |  |  |  |
|  |  |  |  |  |  |  |
| **Multivitamin supplementation** |  |  |  |  |  |  |
| No | 1.8 | (0.9–3.7) | - | - | - | - |
| Yes | 1.0 | (Ref)† |  |  |  |  |
| **Fresh fruits** |  |  |  |  |  |  |
| No | 19.3 | (11.0–33.7) | 8.7 | (4.5–16.6) | 11.6 | 2.8–48.2 |
| Yes | 1.0 | (Ref)† | 1.0 | (Ref)† | 1.0 | (Ref)† |
| **Fresh vegetables or salads** |  |  |  |  |  |  |
| No | 3.2 | (1.6–6.3) | - | - | - | - |
| Yes | 1.0 | (Ref)† |  |  |  |  |
| **Fast food** |  |  |  |  |  |  |
| No | 1.0 | (Ref)† |  |  |  |  |
| Yes | 8.0 | (4.7–13.6) | - | - | - | - |
| **Candy** |  |  |  |  |  |  |
| No | 1.0 | (Ref)† |  |  |  |  |
| Yes | 4.1 | (2.5–6.6) | - | - | - | - |
| **Potato chips** |  |  |  |  |  |  |
| No | 1.0 | (Ref)† |  |  |  |  |
| Yes | 3.2 | (1.9–5.2) | - | - | - | - |
| **Sweetened chewing gum** |  |  |  |  |  |  |
| No | 1.0 | (Ref)† |  |  |  |  |
| Yes | 2.3 | (1.2–4.7) | - | - | - | - |
| **Fresh juice** |  |  |  |  |  |  |
| No | 10.4 | (6.2–17.2) | 3.4 | (1.8–6.5) | - | - |
| Yes | 1.0 | (Ref)† | 1.0 | (Ref)† |  |  |
| **Flavored juices** |  |  |  |  |  |  |
| No | 1.0 | (Ref)† |  |  |  |  |
| Yes | 1.7 | (0.9–3.1) | - | - | - | - |
| **Soft drinks** |  |  |  |  |  |  |
| No | 1.0 | (Ref)† | 1.0 | (Ref)† | 1.0 | (Ref)† |
| Yes | 13.9 | (8.1–23.8) | 5.4 | (2.9–10.1) | 5.3 | 1.5–18.0 |
| **Fresh milk** |  |  |  |  |  |  |
| No | 2.6 | (1.6–4.3) | - | - | - | - |
| Yes | 1.0 | (Ref)† |  |  |  |  |
| **Flavored milk** |  |  |  |  |  |  |
| No | 1.0 | (Ref)† | 1.0 | (Ref)† | 1.0 | (Ref)† |
| Yes | 2.5 | (1.5–4.2) | 4.1 | (2.1–7.8) | 7.7 | 2.6–23.0 |

*The variables excluded based on stepwise selection process using multivariate logistic regression were marked as ‘-’. The variables selected were significant at p-value less than or equal to 0.05.

† Reference category for the variable.

††The variable – ‘Age of the child when breast feeding was stopped’ was excluded to address the issue of collinearity in the final model.
